# Using the Neuroadaptagen KB200z™ to Ameliorate Terrifying, Lucid Nightmares in RDS Patients: the Role of Enhanced, Brain-Reward, Functional Connectivity and Dopaminergic Homeostasis

**DOI:** 10.17756/jrds.2015-006

**Published:** 2015-03-05

**Authors:** Thomas McLaughlin, Kenneth Blum, Marlene Oscar-Berman, Marcelo Febo, Zsolt Demetrovics, Gozde Agan, James Fratantonio, Mark S. Gold

**Affiliations:** 1 Center for Psychiatric Medicine, North Andover, MA, USA; 2 Department of Psychiatry and McKnight Brain Institute, University of Florida, College of Medicine, Gainesville, FL, USA; 3 Department of Clinical Medicine, Malibu Beach Recovery Center, Malibu Beach, CA, USA; 4 Department of Addiction Medicine, Victory Nutrition International, LLC., Lederoch, PA, USA; 5 Community Mental Health Institute, Center for Clinical & Translational Science, University of Vermont and Department of Psychiatry, University of Vermont College of Medicine, Burlington, VT, USA; 6 Dominion Diagnostics, Inc. North Kingstown, RI, USA; 7 Center for Genomics and Applied Gene Technology, Institute of Integrative Omics and Applied Biotechnology (IIOAB), Nonakuri, Purbe Medinpur, West Bengal, India; 8 Center for RDS Research, Victory Nutrition, LLC, Austin, TX, USA; 9 Department of Nutrigenomics, RDSolutions, Inc., Salt Lake City, UT, USA; 10 Department of Personalized Medicine, IGENE, LLC., Austin, TX, USA; 11 Departments of Psychiatry, Anatomy and Neurobiology, and Boston VA and Boston University School of Medicine, MA, USA; 12 Department of Clinical Psychology and Addiction, Institute of Psychology, Eötvös Loránd University, Hungary; 13 Divisions of Addiction Services, & Applied Clinical Research & Education, Dominion Diagnostics, LLC, North Kingstown, RI, USA; 14 Departments of Psychiatry & Behavioral Sciences at the Keck, University of Southern California, School of Medicine, CA, USA; 15 Director of Research, Drug Enforcement Administration (DEA) Educational Foundation, Washington, D.C, USA

**Keywords:** Putative natural dopamine agonist, KB200Z, Functional brain connectivity, Lucid dreams, Nightmares, PTSD

## Abstract

**Background:**

Lucid Dreams are a form of dream life, during which the dreamer may be aware that he/she is dreaming, can stop/re-start the dreams, depending on the pleasantness or unpleasant nature of the dream, and experiences the dream as if he/she were fully awake. Depending on their content, they may be pleasant, un-pleasant or terrifying, at least in the context of patients, who also exhibit characteristics of Reward Deficiency Syndrome (RDS) and Posttraumatic Stress Disorder (PTSD).

**Case Series:**

We present eight clinical cases, with known substance abuse, childhood abuse and diagnosed PTSD/RDS. The administration of a putative dopamine agonist, KB200Z™, was associated with the elimination of unpleasant and/or terrifying, lucid dreams in 87.5% of the cases presented, whereas one very heavy cocaine abuser showed a minimal response. These results required the continuous use of this nutraceutical. The lucid dreams themselves were distinguishable from typical, PTSD nightmares insofar as their content did not appear to reflect a symbolic rendition of an originally-experienced, historical trauma. Each of the cases was diagnosed with a form of RDS, i.e., ADHD, ADD, and/or Tourette’s syndrome. They all also suffered from some form of Post-Traumatic-Stress-Disorder (PTSD) and other psychiatric diagnoses as well.

**Conclusion:**

The reduction or elimination of terrifying Lucid Dreams seemed to be dependent on KB220Z, whereby voluntary stopping of the agent results in reinstatement of the terrifying non-pleasant nature of the dreams. Following more required research on a much larger population we anticipate confirmation of these seemingly interesting observations. If these results in a small number of patients are indeed confirmed we may have found a frontline solution to a very perplexing and complicated symptom known as lucid dreams.

## Background

While Freud [1] regarded dreams as a means of “wish fulfillment” and their understanding by the psychoanalyst as “the royal road to the unconscious,” *nightmares* have been regarded by many as a psychic means of processing threatening information, which cannot be brought into consciousness, without increased anxiety [2]. More recent authors have opined that prolonged or intense nightmares, which involve fearful stimuli, result in reduction of physiological responses associated with these stimuli and/or a reduction in anxiety explains the persistence of some dreams [3].

On the other hand, lucid dreams, as described by the American Association of Sleep Disorders [4], are vivid and life-like and may be associated with an awareness that one is dreaming and/or an ability to control the onset or offset of the dream. The teaching of lucid dreaming is, in fact, a method of overcoming nightmares. Furthermore, there is currently some controversy as to whether lucid dreaming may, in fact, represent a brief, wakeful state [5].

In our sample, the dreams reported were uniformly terrifying in their content. Since some of the patients were also diagnosed with Post-Traumatic Stress Disorder (PTSD), a distinction must be made as to whether “lucid dreams” or the “PTSD dreams” (nightmares) were ameliorated by the use of KB220Z?

Thus, the question posed by these findings is: “Do patients with RDS diagnoses, who report terrifying and/or unpleasant, lucid dreams have their dreams ameliorated by a trial of KB220Z”? [6].

It seems future analyses of lucid terrifying dreams, in this population, should focus on the *symbolic nature* of the dream content and its relevance to the PTSD trauma. If the dreamer is terrified by dinosaurs, for example, this content would not be consistent with that of a trauma-induced dream and could not easily be interpreted as an attempt by the Post-Traumatic Stress Disorder, historical trauma). With these caveats in mind, the authors discuss the nature of dreaming as well as of traumatic dreams and leave aside the question of the origin and function of terrifying, lucid dreams.

In another paper we have discussed the literature related to lucid dreams and provided two cases that showed complete elimination of Lucid dreams with KB220Z [7]. While we do not want to reiterate the same description it is suggested that the following references will serve as a good resource related to Lucid Dreams and neurotransmitter function [2, 8–30].

## Standard Treatment Options for Nightmares

In an attempt to provide medical guidelines to treat unwanted “bad dreams,” especially, in PTSD patients, Aurora *et al.* [19] recommended the following treatment options [see table 1]:

Based on the clinical experience of one of us (TM), it was decided to incorporate a well-researched putative natural safe dopamine agonist, known as KB220Z [31], in a diagnosed PTSD and RDS patient’s treatment plan to determine if this complex would provide any beneficial effects to combat reoccurring lucid nightmares (“bad dreams”), otherwise refractory to other pharmaceutical agents.

In the current case series, we describe patients, who report lucid dreams, which are mainly unpleasant and/or terrifying. In one particular case whereby the patient had a partial response to KB220Z, we provided a more in depth (session by session description) because the patient presented with very high abuse of cocaine. Rendering the scientific question even more problematic is that these patients have all suffered some form of psychological trauma and as such epigenetics (untested) was most likely involved in the promotion of terrifying lucid dreams.

## Methodology and Intake

A standard psychiatric Intake Evaluation was performed on each patient. The categories of clinical information are listed in the case description of each patient. An awareness of the neuro-psychiatric effects of RDS informed the Intake Evaluation and subsequent Psychopharmacological follow-up visits.

Since there may be a much higher prevalence of RDS diagnoses in Suboxone^®^-treated patients, these individuals comprised an important part of our sample. The ubiquitous nature of RDS symptoms and signs, however, is manifested in a general psychiatric sample as well. This clinical experience led to the inclusion of such patients in our sample. The diagnostic criteria in DSM-IV and V were the basis for major psychiatric diagnoses. Patients whose history suggested the presence of RDS-based symptoms underwent an in-depth exploration of their dream life. To this extent, the mental status evaluation of each patient was much more extensive than that of the standard psychiatric evaluation. Sleep-related information, such as myoclonic jerks, sleep-paralysis and periodic leg movements of sleep were also included in the evaluations, as indicated.

Patients were, initially asked to categorize their lucid dreams as either “pleasant” or “unpleasant”, according to their subjective rating. If unpleasant, the patients would tend to use words like, “terrifying”, to further elaborate on their emotional experience of the dreams. No formal rating scales were employed during this preliminary, observational study.

In terms of the graphic displays we arbitrarily appointed a 100% to designate “nightly Terrifying Dreams” as pre-KB220Z baseline. The post-KB220Z result has been presented as percentage decrease from the 100% baseline.

## Presentation of Cases

The following information will describe in clinical terms our experience with each individual case for an understanding of the patient presenting with symptoms especially lucid dreams.

### Case one (Suboxone^®^ patient)-KB

#### Identifying information

The patient is a 36-year-old single male, living with his girlfriend. He was transferring his care, having been detoxified and prescribed Suboxone^®^.

#### Trauma history

He stated that he was sexually abused by a neighbor as a child, where the perpetrator was about three years older than him.

#### Family history

The patient’s mother has suffered from panic attacks and a cousin exhibits blepharospasm. His father was a heavy drinker and died of colon cancer. He remembers him as being “hyperactive”.

#### Social history

The patient is un-married and has no children. He earned a Bachelor’s Degree in Political Science. He had worked as a high school teacher but was currently unemployed.

#### Medical history

The patient denied any history of AIDS, hepatitis, heart disease, hypertension, diabetes or thyroid disease or any other major medical problems, with the exception of obesity. There are no known allergies.

#### ADHD history

When in grade school, he remembers himself as highly distractible and restless.

#### Substance abuse history

The patient first began using opiates at age 31. Prior to that, he had been dependent on alcohol for fifteen years. He had been able to stop his use of alcohol four or five times. He denied any history of withdrawal symptoms, delirium tremors (TSs) or seizures. He stated at the present time, he had no cravings, although he had already been detoxified and was on Suboxone^®^.

He had a gambling addiction for five years, involving poker and race track. He started using alcohol at age 13 and was dependent on it until age 32. He began using Percocet four to five years prior to his admission to this clinic. The first time he used Percocet, he felt “relaxed and calm”. He denied the use of heroin, cocaine, PCP, methamphetamine, or mescaline. He used LSD twice (with no bad trips) as well as ecstasy two or three times a week for two and a half years.

#### Mental status examination

On examination, he was alert and oriented in all three spheres. He was cooperative, and his mood was euthymic. There was no evidence of psychosis, suicidal or homicidal ideation. He denied panic attacks, generalized anxiety symptoms, or obsessive compulsive disorder. His appetite was normal. He reported a remote history of anxiety and depression.

#### Dream life

Patient reports a history of vivid dreams that he could voluntarily control. Their onset was in childhood. He described them in the following way: “It is as if I’m fully awake”. The dreams were terrifying in nature and occurred on a nightly basis.

#### KB220Z Response

**Figure 1:**
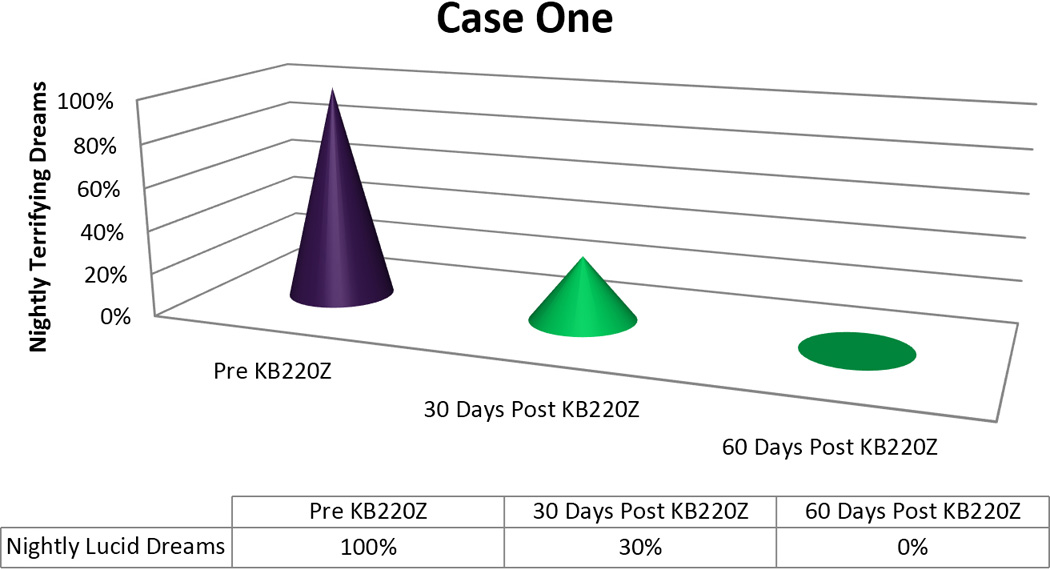
KB220Z response to ameliorate lucid dreams.

He agreed to a trial of KZ200Z, 2 tablets a day. After one month (standard Suboxone^®^ maintenance schedule), he stated his terrifying, lucid dreams, while still occurring nightly, had been reduced in their negative, emotional intensity by 70%. After a second month, the terrifying dreams had completely ceased. During the third month after KZ200B treatment, the patient reported that he had gone off the KZ00B for about 3–4 weeks and had experienced “a mixture of good and bad dreams”. He, then, resumed the regular regimen of KZ200B and no longer had any terrifying dreams [Figure 1].

### Case two (Suboxone^®^ patient)-RM

#### Identifying information

The patient is a 27-year-old, single male, living with his parents.

#### Trauma history

The patient states that his paternal-grandfather would often French kiss him and squeeze his buttocks. His grandfather committed incest with him, between the ages of 8 and 10.The patient states that he was verbally abused by his mother and sexually abused by a 17-year-old neighbor, when he himself was 5 or 6. He reports that he told his parents about this abuse.

#### Family psychiatric history

The patient was adopted at three days of age. His mother was an older teenager at 18, when she gave birth to him. His adoptive family includes a father who suffers from depression, a non-biological brother, who suffers from anorexia and alcoholism and a mother who is described mainly as “manipulative”.

#### Family history

The patient’s father is 89-years-old and demented, the diagnosis having been done not by a neurologist but by his primary care doctor. The patient has always been emotionally close to his father. The patient currently lives with his brother, with whom he has an “odd relationship”. He states they’re both totally dependent on each other and “enmeshed”. His father does all the patients’ Activities of Daily Living and is not on any dementia medications.

When the patient was a boy, his father would engage in activities with him and was affectionate. He had worked as a dentist and, then, had suffered some type of nervous breakdown, of an unspecified nature. He states his father takes B^12^ shots. The patient states his mother died at age 83, of stomach cancer. He notes that she was “irritable and sullen”. She engaged in activities with him as a child but was unaffectionate.

#### Social history

The patient has a Bachelor’s Degree in Food Service. He’s been married twice, the first time for six years, without any children, and the second time, for sixteen years, with teenage step-children, aged 22 and 24.

#### Past medical history

The patient denied any history of major medical problems, including heart disease, hypertension, diabetes, liver disease, asthma, or thyroid disease. He suffered from obesity.

#### ADHD history

During grade school, the patient described himself as spacey and dreamy. RM said he was restless and “the class clown,” who would constantly talk to neighboring students.

#### Substance abuse history

The patient first used drugs at 17, i.e., marijuana. Then, at 21 years of age, he suffered a back injury and became addicted to Percocet. Other abused substances include: heroin, OxyContin, and Percocet. He denied any history of alcohol abuse or dependence. He reported a life-threatening episode, which helped him to “stay clean”.

#### Additional addiction history

The patient admitted to both a sexual addiction and a food addiction. He had a history of a gambling addiction and a frequent urge to steal.

#### Mental status examination

On examination, he was alert and oriented in all three spheres. He admitted to a depressed mood and reported anxiety and agoraphobia. He denied suicidal or homicidal ideation as well as psychotic symptoms or any past history of mania. He admitted to obsessive compulsive tendencies, such as, washing his hands for thirty minutes, multiple times a day as well as an impulse disorder, involving picking his skin.

#### Dream life

The patient reports he has vivid, lucid dreams once or twice nightly. Their onset was in childhood. He dreads going to sleep. He finds himself terrified and prone to sleep paralysis. His dreams are always unpleasant and terrifying. He is unaware of an ability to control his dreams nor of being aware that he is dreaming, while so dreaming.

#### KB220Z response

**Figure 2:**
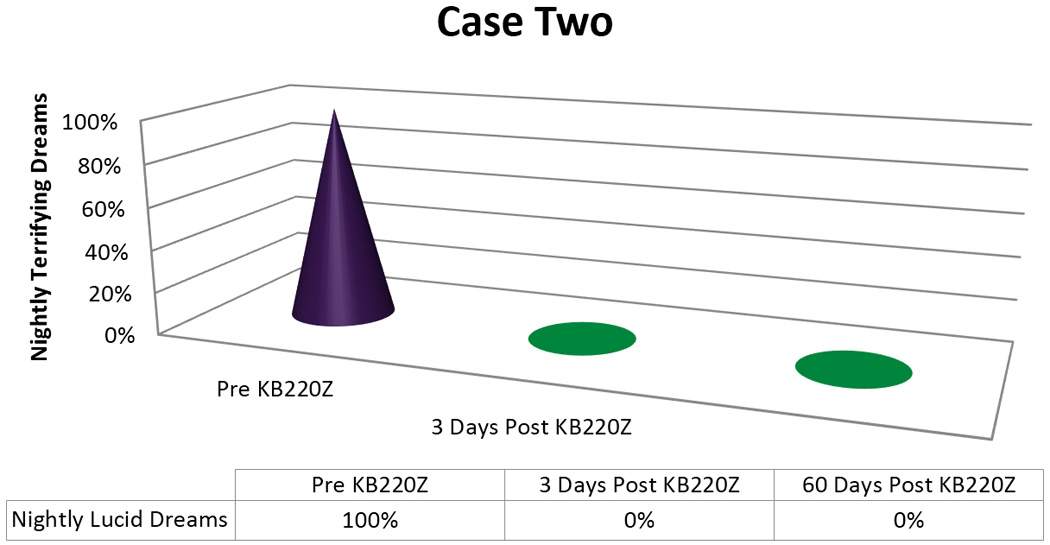
KB220Z response to ameliorate lucid dreams.

The patient was started on KZ200B 2 tablets twice a day. He reported more energy during the day and had no memory of any dreams, terrifying or otherwise, after three days of use [Figure 2]. He continues to use KB220Z with continued benefit related to terrifying dreams.

### Case three (Suboxone^®^ patient)-JN

#### Identifying information

The patient is a 32-year-old, white, divorced mother of one child, diagnosed with Pervasive Developmental disorder.

#### Trauma history

The patient stated she was sexually abused by her stepfather from ages 9 to 11 and also suffered physical abuse at his hands. She stated she was raped and abducted by a friend, at 24 years of age. She stated that that perpetrator is serving thirty-five years in jail. Mother was alcoholic and physically abusive, when intoxicated.

#### Family history

Parents are divorced. At 11 years of age, her mother ‘kicked the kids out” and she went to live with her father. Mother was inattentive and not affectionate, during her early childhood. Father was caring and attentive and is a current emotional support.

#### Family psychiatric history

Mother suffered from alcoholism.

#### Social history

The patient completed nursing school and, then, worked as a children’s teacher. She is the divorced mother of one son, with Pervasive Developmental Disorder (PDD). She was unemployed at the time of her Intake evaluation.

#### Past medical history

History of ovarian cysts, endometriosis. Status-post tonsillectomy.

#### Allergies

Morphine

#### ADHD history

The patient described herself as spacey and dreamy, as well as restless, during grade school. She also reported a history of cutting as a child and was very oppositional during this period as well.

#### Substance addiction history

Patient started using Vicodin at age 14 and was started on Methadone at age 22. After being on Methadone, she was switched to Suboxone^®^.

#### Additional addiction history

She admitted to being a shopaholic but denied alcohol or glucose addiction. She admitted to being a workaholic. She denied gambling but stated she had been a heavy smoker in the past.

#### Mental status examination

On examination, she was oriented in all three spheres, pleasant and cooperative. Her mood was depressed. She denied suicidal or homicidal ideation as well as psychotic symptoms. She denied a recent or remote history of mania. She admitted to generalized anxiety as well as obsessive compulsive disorder symptoms, such as, the persistent need to check the doors and windows of her house frequently. Her appetite was normal but her sleep was disturbed.

#### Dream life

The patient admitted to dreams about being abducted as well as vivid lucid dreams, which occurred nightly. Their onset was in childhood. She stated that these dreams were “beyond terrifying”. They began about three years ago. She stated she avoided sleep, because of them but, in the past, had had a history of pleasant, vivid, lucid dreams. She also described what sounded like “day mares,” wherein she would experience terrifying images, while awake.

#### KB220Z response

The patient was started on KZ200b 2 tablets a day. (Her therapist, who also had a history of RDS behaviors and symptoms, urged her to ask for the supplements, since he himself had been able to alleviate his unpleasant lucid dreams with their use).

**Figure 3:**
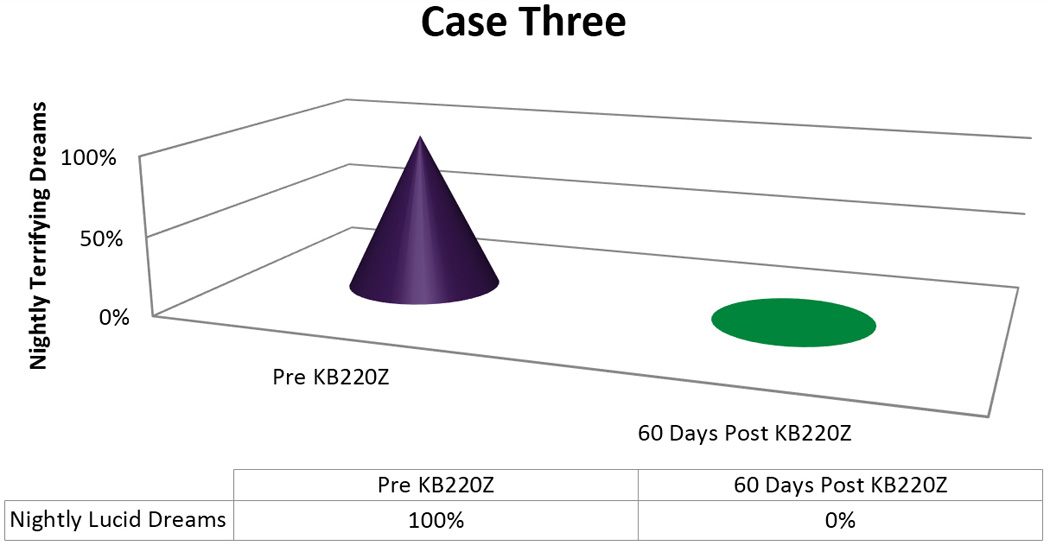
KB220Z response to ameliorate lucid dreams.

Since her provider was not treating her psychiatrically nor prescribing Suboxone^®^, her next follow up visit was in two months, rather than at a shorter interval. At this time, she reported that, she no longer remembered her dreams, with the exception of a “nightmare” related to an up-coming court appearance concerning a custody case. She stated she was completely free of vivid, dreams and had, in fact, reduced her dosage to 3 tablets a day, with the same, salutary effect [Figure 3]. The patient was taking KZ 200Z for two months and continues to take the supplement to the present.

### Case four (depression)-PS

#### Identifying information

The patient is a 58-year-old woman, whose chief complaint was depressed mood and angry outbursts. She was living with her daughter and the latter’s four children.

#### Trauma history

The patient described extensive, physical and sexual abuse as a child.

#### Family psychiatric history

Mother suffered from a cocaine addiction. She never knew her biological father.

#### Family history

She was the daughter of a prostitute, who neglected and was unaffectionate to her, during her childhood. She stated she was the product of an encounter between a famous political figure and her mother. Her justification for the conclusion about her paternity seemed credible.

#### Social history

The patient was a high school graduate and divorced mother of a 33-year-old daughter, with whom she was living. The latter had 4 small children and would expect the patient to care for these children by herself much of the time. The patient and her daughter both received no financial support from the father of their children. The patient and her daughter both supported themselves via disability insurance. The father of the patient’s grandchildren was incarcerated.

#### Past medical history

Obesity, hypothyroidism.

#### Allergies

None

#### Substance addiction history

She admitted to a remote history of alcohol dependence as well as a current smoking addiction.

#### Mental status examination

On examination, the patient was alert and oriented in all three spheres. She was pleasant and cooperative. She complained of severely depressed mood but denied suicidal or homicidal ideation as well as delusions, hallucinations or periods of mania. She admitted to panic attacks and provided a history consistent with ADHD, inattentive type. She denied symptoms of Obsessive Compulsive Disorder (OCD). She complained of poor appetite and insomnia, awakening every two hours-a sleep pattern which had existed for the past year. She states she was not short of breath, sweating or particularly panicked during the night. She complained of daily flashbacks of previous trauma, involving sexual and verbal abuse as a child.

#### Dream life

During the course of treatment, the patient complained of insomnia. She stated that she would awaken every two hours and has done so for the past year. When asked to elaborate on the nature of her dreams, she stated that she felt as if she were fully awake and attentive to their content “to the last detail”. She describes them as nightly “vivid” dreams, which are “unpleasant”. Sometimes, she thought she could actually “hear her neighbors having sex” or “would experience the house as if it were falling down.” She noted that she had a history of being able to wake herself up from such lucid dreams, which were almost always “unpleasant”. The onset of her lucid dreams was about 6 years of age. They were later described as frequently “terrifyingly raw”, involving, for example, “being in sexually uncomfortable situations”. She also dreamt of “being on drugs, smelling her own menses, and fearing that other people could smell her menses.” She denied ever having “pleasant (lucid) dreams.” (In the patient’s opinion, treatment with prazosin made her dreams worse).

#### KB220Z response

**Figure 4:**
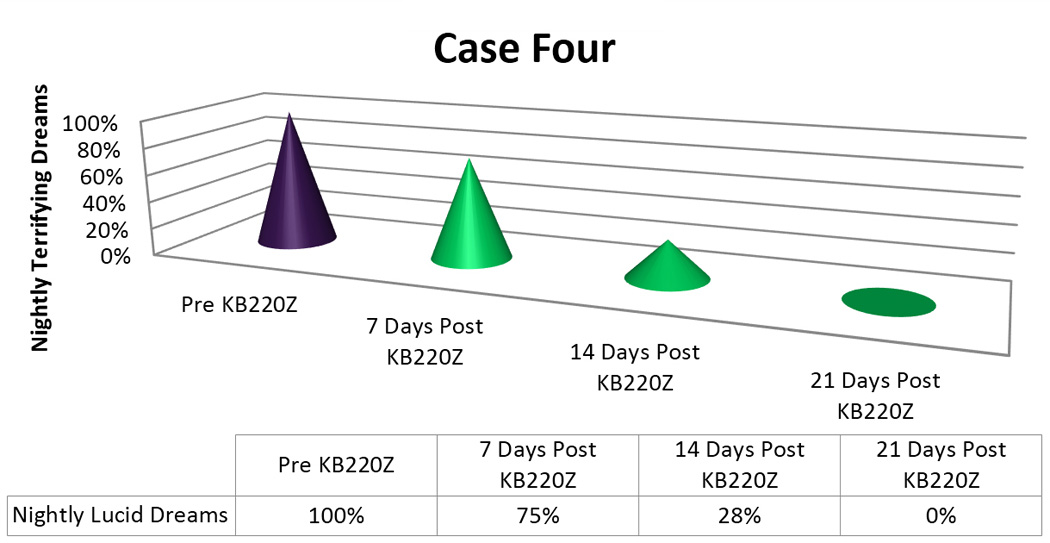
KB220Z response to ameliorate lucid dreams.

She was given KZ200B, two tablets twice a day. After one week, she reported her nightmares had decreased to every other night (vs. nightly). The dreams were still lucid and unpleasant but after two weeks began to occur only twice a week. Interestingly, the ***outcome*** of the bad dreams initially tended to became more positive, insofar as the patient had become *less helpless* and would find herself able to “hold her own in a fight,” in the dream, for example. In addition, after three weeks, the raw, terrifying, lucid dream had completely been eliminated [Figure 4]. (The patient had also lost 12 pounds and was also feeling an “anti-stress effect” from the supplements).

### Case five (Suboxone^®^ case)-BO

#### Identifying information

The patient is a 47-year-old male who lives with his wife and their three children.

#### Trauma history

The patient states that his mother would walk around the house semi-naked in front of the children and family. The patient denies any other history of trauma.

#### Family psychiatric history

Mother suffered from multiple addictions, including alcohol.

#### Family history

Mother was inattentive and unaffectionate during his childhood. Father was attentive.

#### Social history

He has been with his wife for twenty-two years and married eighteen years. He works full-time as a maintenance supervisor for his local city government.

#### Past medical history

The patient denied any major medical problems, including cardiac disease, thyroid disease, asthma, diabetes, or hypertension.

#### Allergies

None

#### ADHD history

During grade school, the patient reported extremely poor concentration and was unable to focus or read books. He was also restless and fidgety.

#### Substance addiction history

The patient first used marijuana at 12 years of age and began using cocaine at 15. At age 27, he started using Percocet, Vicodin, and OxyContin. When asked how he felt, when using the opiates, he stated “On top of my game”. He was a heavy smoker and had been on 16 mg of Suboxone^®^ for seven years, and, at times, had relapsed due to cravings. The patient had a history of heroin use and had used cocaine about 5,000 times over 15 years. He used ecstasy for ten years, beginning at age 12. For twenty-seven years, he used Vicodin, Percocet and OxyContin. He has been on Suboxone^®^ for seven years, with occasional relapses.

#### Additional addiction history

The patient admitted to a current smoking addiction of greater than two packs a day

#### Mental status examination

On mental status examination, the patient was oriented in all three spheres. He denied depressed mood as well as suicidal or homicidal ideation. He also denied delusions as well as hallucinations or of manic episodes. The patient admitted to OCD tendencies and stated that his appetite was normal but his sleep was disturbed.

#### Dream life

The patient admitted to lucid dreams, which are 80% “unpleasant” (examiner’s initial descriptive category see methods). In early childhood, such dreams, had occurred nightly but, more recently, happened two to three times a week.

#### KB220Z response

**Figure 5:**
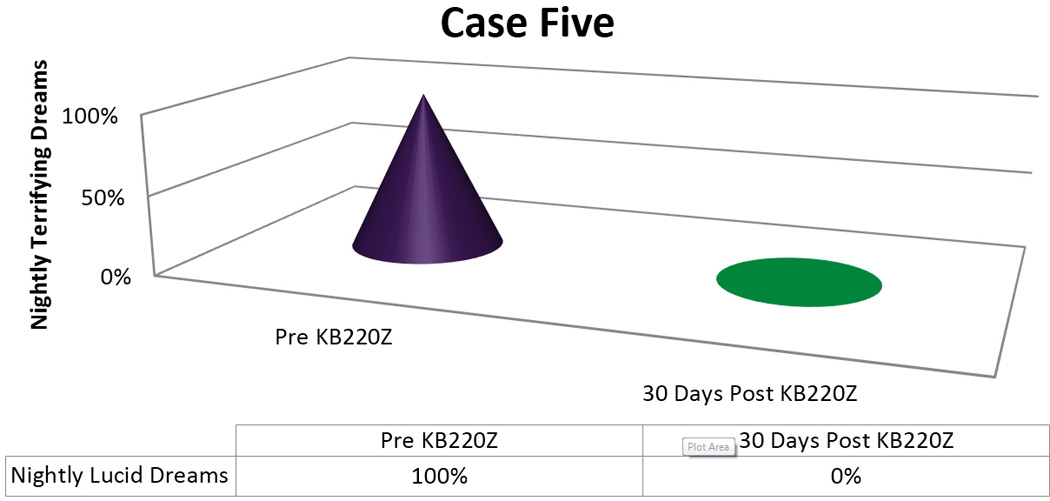
KB220Z response to ameliorate lucid dreams.

After four weeks (standard Suboxone^®^ maintenance, treatment schedule) of taking the KB200Z, his dreams had become pleasant and were no-longer vivid or terrifying [Figure 5]

### Case six (Suboxone^®^ case)-KE

#### Identifying information

The patient is a 42-year-old male currently living at a residential house

#### Trauma history

The patient reports a history of sexual abuse as a child by his “fake uncle,” a nephew of his mother’s female lover. He also states that he was physically and emotionally abused by his mother and her female lover.

#### Family psychiatric history

Mother was reportedly sex-addicted but had no formal psychiatric diagnosis. The patient never knew his biological father.

#### Family history

The patient never knew his father and had tried to find him, without success. He stated that his mother had never told his father that she was pregnant. The pregnancy occurred, when she was in her late 20s. His mother’s girlfriend also had a history of alcoholism, obesity and rage attacks. He stated that his mother frequently was a “follower of her girlfriend’s rage”, viz., mainly, towards him. He stated that his girlfriend’s lover was also promiscuous with both men and women. The patient’s maternal grandfather and grandmother were caring and nurturing. He spent summers at their house. His mother left home, when he was 16 years old, in order to work in a factory.

#### Social history

The patient is separated from his ex-wife and children. He is a high school graduate, who is currently unemployed.

#### Past medical history

The patient suffers from asthma, is Status Post (S/P) motor vehicle accident, with a skull fracture and cerebrospinal fluid leakage. He has sleep apnea but does not use a Continuous Positive Airway Pressure (CPAP) mask. He is diagnosed with epilepsy, which is treated with Depakote.

#### Substance addiction history

The patient used cocaine more than 10,000 times. He also has used angel dust; methamphetamine (four hundred times); heroin (on one occasion, intravenously).

#### Mental status examination

The patient was alert and oriented in all three spheres. He was depressed, rating his mood as 7.5/10 in severity. He denied suicidal or homicidal ideation. He reported auditory hallucinations, which are demeaning in their content. He denied delusions, obsessive compulsive disorder symptoms, or mania. He reported nightmares, related to his history of having found his girlfriend, after she had hanged herself. He reports flashbacks, involving his girlfriend’s hanging as well as his own sexual abuse as a child. He eats three meals a day but his sleep is problematic.

#### Dream life

The patient reported, lucid dreams, whose onset was in childhood at 5 years of age and which tended to occur four to five times a week. He had dreaded going to sleep, because of their terrifying nature. The frequency of these dreams had increased to nightly. Their current content appears to relate to his numerous traumas, especially, his verbal and abuse at the hands of his mother and her lover as well as history of having found his girlfriend after her suicide by hanging. Their vivid, lucid nature, is however, not typical, in the clinical experience of the one of the authors (TM).

#### Response to KB200Z

The patient was started on one KB200Z tablet twice a day, in addition to his psycho-pharmacological regimen, in an attempt, based upon two prior serendipitous patient experiences to ameliorate his terrifying, lucid dreams [7]. This lower than advised starting dose was used, because of the presence of medication-refractory, auditory hallucinations. In addition, there was no expectation that this regimen would impact his daytime flashbacks, which did, in fact, occur.

Since this patient represents a very heavy cocaine abuser we present an extended detail tracking of his response to KB220Z unlike the other five cases.

##### Session I (Two KB200Z tablets a day)

After one week, the patient stated that he continued to have “nightmares” every night, but they were somewhat “more nostalgic”. They had been described as realistic and as extremely lucid, as originally. He states that they were uniformly unpleasant and they involved post-traumatic events, such as, being beaten by his mother and his mother’s girlfriend as well as finding his own girlfriend dead by hanging. He also reported flashbacks on a daily basis, involving finding his girlfriend. He stated that they could occur “either all day long or “once a day”. He stated that he would still “reach out his arms in a “life-like manner” and would “wake up with his arms extended”. He stated that these dreams would involve “grabbing for her” and he would find himself waking up, reaching out for her.

##### Session 2 (Three KB200Z tablets a day)

He stated, with respect to his dreams, that he had “wicked,” vivid dreams, some of which were now pleasant and some unpleasant. He stated that he could re-start the good dreams.

##### Session 3 (Three KB200Z tablets a day)

The patient stated that he was still having vivid lucid dreams and was now using his CPAP mask for his sleep apnea. He had lost some weight, due to increased walking. He stated that his unpleasant dreams were now occurring four to five times a week.

##### Session 4 (Two KB200Z tablets a day)

The patient stated he was sleeping seven hours but that there had been no change in his lucid dreams, whose frequency was, once again, nightly. The dreams seemed to be less vivid but continued to be “unpleasant” and involved images of being bitten by his mother and of having found his girlfriend. With respect to more classic, post-traumatic stress disorder symptoms, he stated that he had *daily flashbacks* of finding his suicide girlfriend but that these had decreased from a daily basis “all day long” to “once a day”.

##### Session 5 (Three KB200Z tablets a day)

The patient next reported that he was now dreaming three to four times a week about his girlfriend (vs. four to five times a week), with no change in the lucidity of the dreams. He continued to dream about his mother and her lover screaming at him but these dreams were now only occurring two to three times a week. They were still lucid but the patient was now able to shut them off, when they became too terrifying. His dreams were now 80% unpleasant vs. 20% pleasant. The pleasant dreams involved camping with his children. At this point, he was placed on KB 200Z, four pills a day. He had been on three a day and he stated that his flashbacks had decreased from occurring “all day long to once or twice a day.”

##### Session 6 (Four KB200Z tablets a day)

Prazosin was decreased to 2 mg a day, because it seemed to be having no effect and because other patients had reported that it aggravated their lucid dreams. His dream life was “still kind of rough”, although he recently dreamt of being in his grandfather’s house, which had been a happy place for him. This kind of dream represented “a new experience” and was occurring about twice a week. His flashbacks continued to decrease to once a day (again, involving finding his girlfriend). His KB200Z regimen was increased to five a day.

##### Session 7 (Five KB200Z tablets a day)

Wakeful flashbacks were now occurring every other day, still involving his girlfriend. He continued to have unpleasant lucid dreams, 50% involving abuse by his mother and her lover and 50% finding his girlfriend. He noted the occurrence of more “happy dreams” with respect to his children, estimating these were occurring about three times a week. He also reported a 60% decrease in the intensity of the unpleasant dreams. His KB200Z regimen was, then, increased to seven a day.

##### Session 8 (Seven KB200Z tablets a day)

With respect to his dream life, the nightmares continued to occur every night, but “not all night long”. He stated that they were “now once a night”. (They always involved his girlfriend, his mother and his mother’s lover). He was now dreaming about his children (when he had more pleasant dreams). The proportion of negative vs. positive dreams was 70 to 30%. His waking flashbacks, only “occurred once in a while” (twice a week or less). The KB200Z regimen was increased to eight tablets a day.

##### Session 9 (Zero KB200Z tablets a day) (Baseline)

The patient had held the KB200Z tablets, on his own volition. His lucid dreams increased to three or four times a week, wherein while taking the tablets, they had decreased from a nightly basis). Without the KB200Z, his dreams were 100% unpleasant (i.e., no happy dreams).

### Case seven (sleep disturbance)-AM

#### Identifying information

The patient is a 22-year-old female, living with her mother and four children. She states her mother urged her to come for treatment. She has suffered from insomnia for three years and sleeps only two to three hours a night. She also suffers from decreased motivation as well as a decreased ability to concentrate and decreased ambition.

#### History

The patient stated that her problems with attention began at about ten years of age, when she was treated for being highly distractible. She was given a trial of Ritalin and wound up “staying up all night”. The patient also sustained burns during her early childhood, when she accidentally turned on scalding, hot water in the bath tub. She had forty-five surgical procedures, as well as a knee replacement to deal with the effects of this trauma. She has had multiple skin grafts as well. She also notes a childhood history of cutting, with non-suicidal intention.

#### Trauma history

The patient denies any history of sexual, physical or emotional abuse but does report verbal abuse by her mother.

#### Family psychiatric history

The patient states that her mother suffers from depression and that her maternal grandmother had “mental issues”.

#### Family history

The mother is 50-years-old and described by the patient as “really angry and someone who gets stressed out easily”. She states her mother was not affectionate towards her nor engaged in any activities with her, when she was about five years of age.

The patient’s father is in his mid-60s, and she describes him as “a cold jerk”. He fathered twelve children but “doesn’t care about any of us”. The patient noted she has twelve siblings, with whom she has only a distant emotional relationship.

#### Social history

The patient states that she attended high school through sophomore year and, then, was expelled, because of fighting. She eventually got her G.E.D. a few years later. She has never been married but has four children, with their father currently in jail.

#### Past medical history

The patient denied any history of any major medical problems including, asthma, seizures, hypertension, or thyroid disease. She suffers from obesity (200 pounds).

#### Allergies

There are no known allergies.

#### Substance addiction history

The patient currently denies any drug or alcohol use. In the past, she drank to intoxication on weekends but never was charged with any DUIs nor did she suffer withdrawal seizures or the DTs. She began drinking at 16 years of age.

The patient states she currently doesn’t use any street drugs but that, in the past, she used marijuana daily for two to three years. She stated that its use made her feel “calm”. She denied any use of cocaine, heroin, ecstasy or LSD.

#### ADHD history

The patient describes herself during grade school as restless and prone to leaving class, talking to her neighbors and touching things. She also stated that she was spacey and would frequently daydream. In addition, she also would violate prohibitions by her mother, when her mother said, for example, “don’t touch that stove!”.

#### Mental status examination

The patient was alert and oriented in all three spheres. She was pleasant and cooperative. She described her mood as depressed, rating it as a 7/10 in severity with 10 being maximal. She denied suicidal or homicidal ideation as well as auditory hallucinations. She admitted to olfactory hallucinations on rare occasions, involving “smelling poop”. She denied delusions as well as any history of manic episodes. She reported a tendency to have obsessive compulsive disorder symptoms. She denied panic attacks and stated that she slept between two and three hours a night, on average. She reported her appetite was fair, eating two meals a day. She stated that she only slept two to three hours a night and did not experience true nightmares.

#### Dream life

The patient stated she had a history of vivid, lucid dreams, which she could re-start, if they were pleasant. The dreams would frequently involve feeling gusts of wind on her arm. She stated that many of these dreams were unpleasant and involved seeing herself among snakes.

A few months into treatment, the patient reported that her lucid dreams were divided, with about 50% being “pleasant” and 50% “unpleasant”. These dreams occurred about once or twice a week. The bad dreams would also involve a “porcelain doll trying to eat me and me trying to scream”. Her pleasant lucid dreams involve sitting on a beach with a breeze blowing on her face.

#### KB220Z response

The patient was started on KZ200B, 2 tablets, twice a day. She stated that, after two weeks, her vivid dreams were now only occurring once a week, and she had had one unpleasant dream she remembered, involving seeing a “worm in my eyeball”.

**Figure 6:**
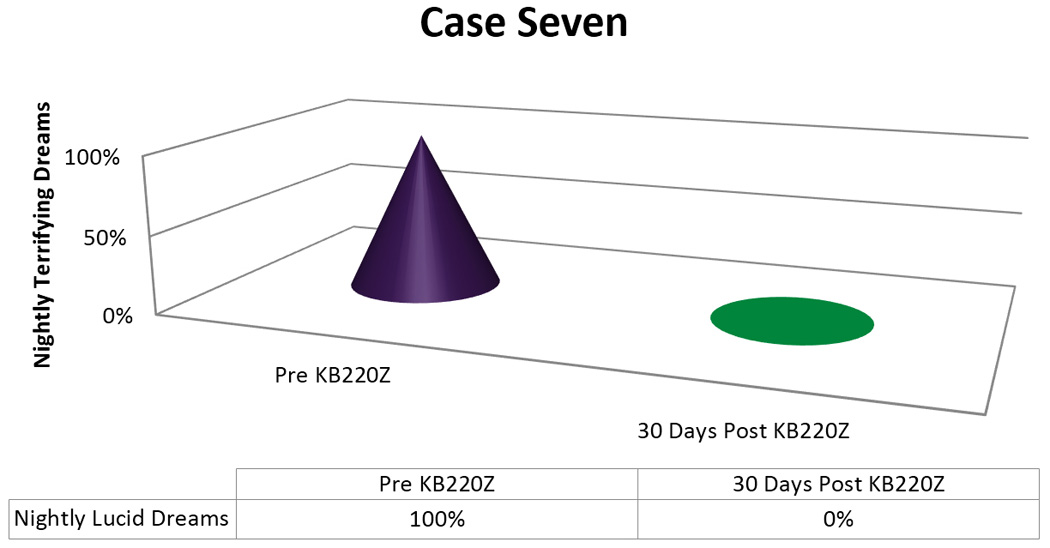
KB220Z response to ameliorate lucid dreams.

When seen a month later, the patient had on her own, reduced her KZ200B tablets to one, twice a day. She reported that she was now able to “sleep eleven hours, with no dreams at all!” The patient’s Dream Life has remained normal, taking one tablet twice a day [Figure 6].

### Case eight (PTSD)-AS

#### Identifying information

The patient is a 33-year-old woman, who lives with her mother and her girlfriend. She reports that she has been treated for Post-Traumatic-Stress-Disorder. She was most recently treated by her primary care provider with psychotropic medications.

#### History

The patient states the onset of her psychological problems occurred at age 14, when she began to remember earlier instances of trauma. She reports she was hospitalized at the age of 16, for being angry and depressed. She was involved in psychotherapy for one year in her early 20’s. She felt the therapy was “somewhat helpful”.

#### Trauma history

The patient states that she was sexually abused at six years of age, on two occasions, both involving intercourse. The perpetrator was a maternal uncle. The patient states she never told anybody and there was no other evidence of abuse. She reports she was physically abused by her father and mother (struck with a belt and hit in the face). She stated both her parents had drug problems.

The patient also reported emotional abuse, when her mother would say to her, “I wish we had never had you, you little fuck”.

#### Family psychiatric history

The patient states that her mother and father were both drug and alcohol addicts.

#### Family history

The patient’s mother is 54-years-old and is described as “always in bed”. When she is not in bed, she is “using drugs”. The patient’s mother was not affectionate nor did she engage in any activities with the patient as a child. The patient states she was raised by her maternal grandmother, from the age of 10.

When asked to describe her father, she stated he was 54-years-old and a “miserable idiot”. He did not engage in any activities with her, when she was young nor was he affectionate.

#### Social history

The patient attended the seventh grade but then was expelled for violent acting out and inattentiveness. She has never married and has no children. She is currently in a lesbian relationship for the past three years. She has not worked in thirteen years and is currently on Social Security Disability.

#### Past medical history

The patient denies any major medical problems, involving hypertension, cardiac disease, thyroid disease, diabetes, or hypertension. She states that she had an MRI of the brain for headaches and that she also had a history of “seizures”.

#### Allergies

The patient developed a rash, when taking codeine.

#### ADHD history

The patient states she was distractible and restless during grade school and also was unable to “remember what I had read”.

#### Mental status examination

The patient was alert and oriented in all three spheres. She was pleasant and cooperative. She described her mood as depressed, rating it as 7/10 in intensity, with 10 being maximal depression. She denied suicidal or homicidal ideation as well as auditory or visual hallucinations. She sometimes had olfactory hallucinations, involving “the smell of urine” and “rotten food”. She denied symptoms of obsessive compulsive disorder, delusions or any history of mania. She reported a history of panic attacks, involving shortness of breath and “tunnel vision”. The latter lasted about five to ten minutes and occurred about two times a week.

The patient ate only one meal a day and stated she only slept three hours a night. She reported that she has “nightmares one or two times a week but denied flashbacks. She also admitted to “vivid lucid dreams” as well as periodic leg movements of sleep.

#### Dream life

Once the patient was stabilized, with respect to her depression and panic, inquiry was made into the nature of her Dream Life. She stated the lucid dreams began during childhood and persisted about three to four times a week, until the present. They are experienced, mostly, as “unpleasant”. She experienced the dreams as if she were fully awake. She would often feel as if she were being drowned or “held upside down”. She reports she has been unable to re-start any of her “pleasant,” lucid dreams nor has she been aware of the fact that she was dreaming, while dreaming

She also reported myoclonic jerks, while falling asleep, and distinguished her lucid dreams from her post-traumatic stress disorder dreams, with the latter involving images of “being molested”, in contrast to her lucid dreams, which involved being “dunked and drowned”. She currently slept only three hours a night. It should be noted that her dreams are of being drowned or dunked upside down, while they appear to be associated with a feeling of helplessness, do not, in their content, symbolize, in the authors’ opinion, the molestation she suffered, on two occasions, at the age of six nor do they seem symbolize the physical abuse she endured, when her parents punished her harshly.

#### KB220Z response

The patient was started on KZ200Z, two tablets, twice a day. In one month, she stated that she was now sleeping five hours a night and could no longer remember any dreams. She also no longer had a “fear of falling asleep”.

**Figure 7:**
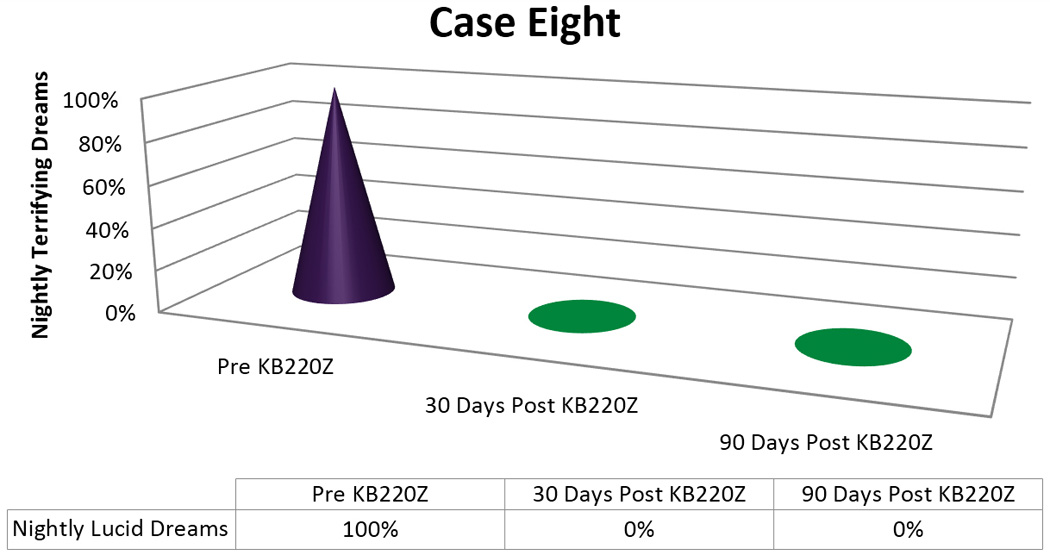
KB220Z response to ameliorate lucid dreams.

About three months later, when the patient was continuing to take the regimen, she stated that she continued not to have any memory for dreams and spontaneously stated “That stuff works good!” Even though she was without the supplements for a period of ten days, her lucid dreams still did not recur [Figure 7].

## Discussion

Importantly, we found in seven out of 8 cases or 87.5% a positive response rate following the addition of KB220Z to these troubled PTSD/RDS diagnosed patients presenting with terrifying Lucid Dreams. While the time for elimination of these dreams varied with each patient [see figures 1–5] in terms of frequency, intensity and nature of these dreams we are encouraged with the clinical outcome. In this case series, we note that in the seven responders the patients had never had an extended period of happy dreams in their lives. It is noteworthy that in Case 6 the patient received partial benefit from taking the KB220Z in that the terrifying dreams were reduced and changed from terrifying to more pleasant dreams with an elimination of unpleasant daytime flashbacks, we cannot explain this lack of response. However, we could suggest that the abuse of cocaine more than 10,000 cocaine times has typically been associated with extensive SPECT brain hypoperfusion in the medial temporal and orbital frontal regions [32]. We believe that while KB220Z had some effect on this patient due to a significant impairment in dopaminergic function (probably due to down regulation of D2 receptors) the activity of KB220Z was masked.

In our opinion, it was the *lucid dreams* that responded to the supplement, since their content, in general, did not seem to reflect a reference to “trauma”. An obvious exception would be the patient, who reported nightly dreams of finding his girlfriend, who had hanged herself. Although this content was consistent with a PTSD nightmare, the fact that his dreams were so intense and never dissipated over time, in terms of their frequency or intensity, argues for the strong admixture of a lucid, terrifying quality to the “traumatic” nature of his nightmares.

As we previously pointed out in an earlier published study showing a similar reduction of terrifying Lucid Dreams in two other cases with diagnosed PTSD/ RDS [7] it would appear that neither powerful dopamine agonism nor noradrenergic A1 nor A2 blockade was responsible for this dramatic effect, since neither Dextro-amphetamine, prazosin, clonidine nor their combination had any apparent effect of the lucidity or unpleasant content of nightly dreams in these cases.

To reiterate there are a number of published papers that describe the ingredients in the KB220Z complex consisting of various precursor amino-acids for synthesis of serotonin, a chromium salt; GABA and dopamine; a known natural enkephalinase inhibitor; a natural benzodiazepine stimulant and a herbal substance known to inhibit COMT and mitochondrial MAO-A [33]. Following 28 clinical trials with KB220 variants, since 1982; there is strong evidence that this complex, which was designed to mimic the “Brain Reward Cascade,” appears to increase dopamine release and activation across the Brain Reward Circuitry [34].

Most recently we published that KB220Z induced an increase in BOLD activation in caudate-accumbens-dopaminergic pathways compared to placebo following 1-hour acute administration in abstinent heroin addicts. KB220Z also reduced resting-state activity in the putamen of abstinent heroin addicts. We also observed that three brain regions of interest were significantly activated from resting state by KB220Z compared to placebo. It is quite possible that in part the finding that KB220Z increased functional connectivity observed in a putative network that included the dorsal anterior cingulate, medial frontal gyrus, nucleus accumbens, posterior cingulate, occipital cortical areas, and cerebellum may have relevance to the current findings [6]. Moreover, Blum et al. [35], using quantitative EEG (qEEG) demonstrated regulation of widespread theta activity within the cingulate gyrus in abstinent, psycho-stimulant addicts with an increase in alpha and low beta activity within one-hour post administration (see Ogilvi et al. [28]). In our unpublished rodent work, on resting state functional connectivity, we clearly demonstrated selective, robust activation of KB220Z vs. placebo in the reward system of the brain. We found that connectivity with regions, such as, the nucleus accumbens, the anterior cingulate, pre-limbic and infra-limbic structures was significantly increased with KB220Z treatment. Moreover, there is also evidence of recruitment of additional brain structures, such as, the hippocampus, anterior thalamus, and somato-sensory regions-which findings could indicate the inclusion of these and other regions into a putative, activated, neural network. Interestingly, evidence of activation at cortical loci, potentially involved in “nightmares” [36] was also demonstrated. Finally dopaminergic agonist therapy may be important for PTSD and RDS behaviors. Willuhn et al. [37], reported that cocaine use and even non-substance-related addictive behavior increases as dopaminergic function is reduced. Chronic cocaine exposure has been associated with decreases in D2/D3 receptors and was also associated with lower activation of cues in occipital cortex and cerebellum, Therefore, treatment strategies, like dopamine agonist therapy, that might conserve dopamine function may be an interesting approach to treating terrifying lucid dreams without powerful agonistic therapy that could down regulate D2 receptors.

## Limitations

The primary limitation to this present work is the very small number of patients. The other limitation is that this is not a double blind randomized controlled placebo study and as such the results must be considered preliminary at best. Another caveat is that there was no standard evaluation of timing following the administration of KB220Z. Since the diagnosis was made at various clinical practices /institutions there is no standard of practice and could lead to spurious results.

## Conclusion

In conclusion, the discovery that KB220Z increases functional connectivity has widespread implications for treatment of psychiatric diseases (e.g. RDS) via its impact on the above brain regions. Such activation could induce and, thus, promote enhanced dopaminergic function-a factor leading to the elimination and/or marked reduction in unwanted, lucid “bad dreams” in PTSD patients, such as, in these reported cases. KB220Z may, by the same reasoning, alleviate other causes of lucid bad dreams [20, 22] and, in this regard, deserves considerable and more intensive investigation. Albeit the very small number of patients we have now confirmed our first observation regarding this important finding [7] but until much larger studies are executed in double-blind fashion these results must remain preliminary.

## Figures and Tables

**Table 1:** Standard treatment for lucid dreams.

Prazosin is recommended for treatment of Posttraumatic Stress Disorder (PTSD)-associated nightmares. Image Rehearsal Therapy (IRT) is recommended for treatment of nightmare disorder. Systematic Desensitization and Progressive Deep Muscle Relaxation training are suggested for treatment of idiopathic nightmares. Clonidine may be considered for treatment of PTSD-associated nightmares. The following medications may be considered for treatment of PTSD-associated nightmares, but the data are low grade and sparse: trazodone, atypical antipsychotic medications, topiramate, low dose cortisol, fluvoxamine, triazolam and nitrazepam, phenelzine, gabapentin, cyproheptadine, and tricyclic antidepressants. Nefazodone is not recommended as first line therapy for nightmare disorder because of the increased risk of hepatotoxicity. The following behavioral therapies may be considered for treatment of PTSD-associated nightmares based on low-grade evidence: Exposure, Relaxation, and Re-scripting Therapy (ERRT); Sleep Dynamic Therapy; Hypnosis; Eye-Movement Desensitization and Reprocessing (EMDR); and the Testimony Method. The following behavioral therapies may be considered for treatment of nightmare disorder based on low-grade evidence: Lucid Dreaming Therapy and Self-Exposure Therapy. Venlafaxine is not suggested for treatment of PTSD-associated nightmares. No recommendation is made regarding clonazepam and individual psychotherapy because of sparse data.
